# Head posture control under perturbed conditions in progressive supranuclear palsy patients

**DOI:** 10.3389/fnsys.2025.1466809

**Published:** 2025-05-26

**Authors:** Vittorio Lippi, Christoph Maurer, Christian Haverkamp, Stefan Kammermeier

**Affiliations:** ^1^Institut für Digitalisierung in der Medizin, Medizinische Fakultät und Universitätsklinikum, Universtität Freiburg, Freiburg im Breisgau, Germany; ^2^Neurozentrum der Uniklinik Freiburg, University of Freiburg, Freiburg im Breisgau, Germany; ^3^Neurologische Klinik und Poliklinik, LMU Klinikum, Ludwig Maximilians Universität, Munich, Germany

**Keywords:** progressive supranuclear palsy, head motion, modeling, feedback control systems, parameters identification, posture control, human motor control

## Abstract

**Introduction:**

In neurodegenerative brain diseases like Progressive Supranuclear Palsy (PSP), clinical studies underscore the crucial role of head motion deficits. Similarly, advanced stage Idiopathic Parkinson’s disease (IPD) is known to display significantly altered posture control and balance patterns involving the head segment.

**Methods:**

This study investigates the relative differences in head control during a perturbed upright stance paradigm between patients affected by PSP and IPD, compared to healthy control subjects using dynamic system modeling. The resulting neural model underlines how PSP primarily affects head control, whereas IPD primarily affects the control of the whole body’s center of mass. A neck control model, based on the hypothesis of modular posture control, is proposed to emulate the PSP data in particular.

**Results:**

A larger passive stiffness was observed for both groups of patients, with eyes closed, suggesting that the head moves together with the trunk. With eyes open, the active proportional gain KP is relatively larger in all cases, indicating that the head is directed closer to the vertical by the visual contribution. Since this was held for all investigated groups, findings support the notion of intact visual contribution to posture control among PSP and IPD despite the impaired supranuclear eye guidance among PSP.

**Discussion:**

The proposed neural model’s characteristics will aid in future patient data analysis, disease progression monitoring, and possible modulation of disease-specific features through therapeutic intervention. For engineering and robotics implementations, uses for strengthened resilience of head stabilization are discussed.

## 1 Introduction

Several neurodegenerative diseases in humans predominantly affect motor control capabilities, particularly stability of stance. Eventual loss of self-stabilization with immobility contributes decisively to consecutive infectious complications and death in these diseases. This study analyzes features of Progressive Supranuclear Palsy (PSP) and advanced stages of Idiopathic Parkinson’s Disease (IPD). Both diseases feature velocity-independent muscle stiffness (rigidity) during passive motion, and slowness (bradykinesia) during active motion with optional resting tremor.

PSP is a rare, rapidly disabling atypical Parkinsonism disorder based on intracellular tau protein accumulation with progressive cell loss focused on mesencephalic structures, where neural centers of vertical gaze control and pathways of axial motor control reside (Iankova et al., 2020; Höglinger et al., 2017). With an onset usually in the sixth or seventh decade of life, patients present falling backward under unprovoked conditions within the first year of disease manifestation, resulting in unprotected injuries from falls, rapid immobilization, and eventual death from immobilization-related complications, like aspiration pneumonia with a median of 6 years life expectancy. By comparison, IPD is a frequent neurological disease slowly progressing over more than a decade, defined by the degeneration of dopaminergic neurons throughout the brainstem and adjoining systems by alpha-synuclein accumulation. In IPD, rigidity, and bradykinesia can be managed well by dopaminergic medication over most of the first decade of the chronic disease. Later, likely due to progressive degeneration of non-dopaminergic systems, falling becomes a relevant issue. Falling in IPD is typically forward during the initiation or termination of gait by either shifting mass forward, but being unable to initiate gait patterns, or by stopping gait without the termination of the forward mass shift. These falls respond poorly to medication and contribute to eventual immobilization and mortality.

Features of IPD stance instability and its clinical features have been studied extensively throughout the literature and can be considered a hallmark of clinical neurophysiology; based on these features, neural models of its deficits have emerged along with neural network modeling as a discipline. PSP stance deficits, however, are poorly understood and ill defined, due to the relative rarity of the disease and the previous assumption of being simply another type of Parkinsonism with exaggerated rigidity predominantly among axial musculature; only in recent years has it become apparent through clinical studies that PSP axial disability disorder involves an active process of exaggerated upper body and head motion to minimal floor instability (Kammermeier et al., 2018; Kammermeier et al., 2024), but with intact proprioceptive sensory input from the neck (Kammermeier et al., 2017). This abnormal head control pattern attempts to align the head along the vertical axis of the tilting floor segment. By contrast, IPD postural control attempt to align the head along the vertical axis of gravity when perturbed.

These different strategies (Kammermeier et al., 2018; Kammermeier et al., 2024) of body and head postural control led us to emulate the respective strategies in a refined neural control model. Previous PSP and IPD patient recording studies, as referenced above, relied on a simplified but well - established proportional derivative integral PDI controller for a two- to three-segmental inverted pendulum model. The objective of this current modeling study was to restructure, refine, and validate a neural model of a head-on-body postural control model for externally perturbed stance based on the available three-dimensional motion capture data of PSP and IPD patients, as well as healthy reference subjects. Disease-defining alterations or defects to a baseline “healthy” model were to be created, from which certain modifications, alterations or defects would elicit deficits specific to either PSP or IPD. Then, the resulting neural model was to be applied to the respective data from PSP and IPD patients for validation.

The differential motion of the head segment relative to the trunk with the center of body mass bears particular importance. The head holds two of the three main systems required for the subjective coordinate system of self-in-space: the vestibular system measuring 3D-angular acceleration and alignment of the head relative to gravity and the visual system allowing self-reference to gravity by indicators of the visual horizon (Lopez and Blanke, 2011; Brandt and Dieterich, 2019). Proprioceptive self-referencing between the individual body segments as the third system is used to translate the vestibular-visual reference system from within the head into a completed coordinate system of the whole body relative to both support surface and space. These sensor systems complement each other by sensor quality and overlapping time resolution features. Free motion of the head with its integrated sensors during motion presents a particular challenge to this integration; it works well in healthy humans, but presents a challenge in which most humanoid robotics have failed. The advantage of an independent “head” appendage to the main body mass with sensor arrays allows for improved situational overview during body motion and the possibility to explore where the whole body would not fit or should remain concealed. This comes with the tradeoff of increased computational demand to coordinate body motion and stabilization based on the externalized coordinate systems (Kammermeier et al., 2024; Kammermeier et al., 2017).

The task of perturbed upright stance by support surface tilt presents a controlled environment challenge of head motion control. External force perturbations acting higher up on the body can be considered to occur more frequently in everyday conditions rather than small-angle support surface tilts. For the paradigm of PSP postural stability studies this design approach was chosen for six particular reasons however:

- Tilt around the ankle joint provides a stimulation mechanism tailored for the inverted pendulum approach of modeling, which is also easily reproducible, continuously applicable and does not rely on encumbering additive force applicators around the torso, in comparison to effectors designed to have an angle of attack near the body’s center of mass.
- The application of force around the ankle propagates the largest challenge of compensation to the most distal element of the multi-segmental pendulum – the head segment – as the object of particular interest due to the senor systems residing there: visual and vestibular.
- The clinical notion of PSP patients falling frequently not to external force action but rather “minimal floor unevenness” or no apparent reason at all from neutral stance. This unexpected unevenness may be simulated in the way of small-angle surface tilt.
- Also, advanced IPD patients experience falls during initiation or termination of gait, in which a relative delta of forward displacement of the upper inverted pendulum aspects translate against the surface. Inversely, this instability can be simulated by relative backward tilt of the surface.
- The small angle challenges provide a safe and limited exposure challenge to severely posturally unstable patients, as demonstrated in the respective experimental studies (Kammermeier et al., 2018; Kammermeier et al., 2024), and as a sandbox study for challenges in engineering and robotics.
- From small tilt angle challenges, the simulated system can be extrapolated to higher angle challenges and compared to the scarcer datasets of patient data that can be obtained in the real-world equivalent.

To examine patients in a clinical context, an improved neural model created on the basis of group population data (PSP, IPD and healthy subjects) would allow for the definition of specific sets of objective disease-characteristic variables to be recorded along the individual progression of the respective disease, in order to describe alterations of certain variables during the natural course of the respective disease and – possibly - to determine changes by future therapeutic interventions of various kinds (e.g., medication, new molecular therapies, electric neurostimulation) beyond mere clinical grading, which remains subject to considerable inter-rater and intra-rater inconsistencies despite all efforts of standardization (Iankova et al., 2020; Goetz et al., 2010). Such neural models would also allow for rapid automated analysis and output of specific variables immediately after recording in an apparative setting, which would aid tremendously in implementing clinical routine measurements.

The study of human posture control and the assessment of patterns associated with pathological conditions can be performed by analyzing posture control as a dynamic system in terms of input (stimuli) and output (body sway). In this work, we propose both a statistical assessment of the differences between healthy subjects and patients (PSP, IPD) based on a direct analysis of the input–output relationship expressed in terms of the transfer function and a study of the parameters obtained by fitting a dynamic model onto the experimental data. These two approaches are complementary: the analysis of the responses can highlight significant effects associated with the diseases, and the model parameters can suggest a hypothesis for a meaningful description of causes behind the observed effect, e.g., changes in joint stiffness or delay in the neural control loop. This approach to disease entities among groups of subjects can be envisaged to be helpful as a tool in diagnostic patient single trials, particularly when followed up along the time domain of months or even years, and as an instrument to study the disease in general based on population data in conjunction with other modalities of disease characterization like imaging and biobanking.

This computational approach has been used successfully in the past. For example, in studies on a specific eye motion control disease, namely downbeat nystagmus (Glasauer and Rössert, 2008), an effective drug treatment could be conceptualized, tested and validated thanks to a computational neural model. The study of posture control may also benefit from a dynamic system approach, which bridges the high level of behavioral observation to a reductionist description of the process.

## 2 Materials and methods

### 2.1 Body sway and frequency response functions

**Figure 1:**
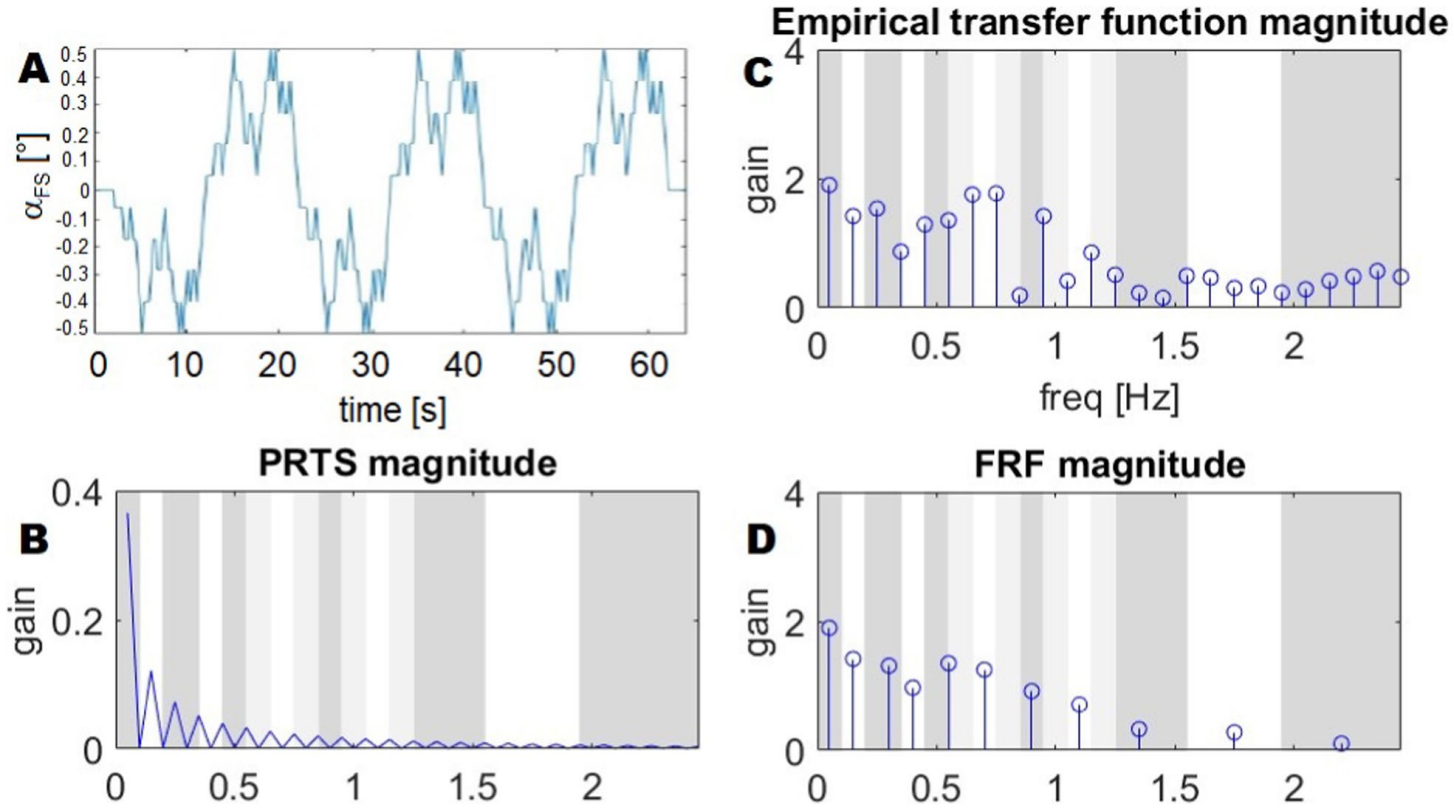
Stimulus profile (PRTS), its spectrum, and example of a FRF. **(A)** The time profile of the PRTS signal, used as reference for the platform tilt. **(B)** The magnitude of the DFT of the PRTS, gain is a unit-less number, as it is the ratio between two angles. **(C)** Empirical transfer function from Goetz et al. (2010). **(D)** FRF resulting from the averaging of frequency bands. The bands on the background show the frequency ranges over which the spectrum is averaged: white and dark grey represent ranges associated with groups of frequencies. The sets of frequencies overlap, with light green bands belonging to both contiguous groups and a sample of the transition between two bands belonging to both groups. As the FRF is averaged in the complex domain, the average shown in the plot is not the average of the magnitudes.

The *frequency response function*, FRF, is an empirically computed transfer function between the stimulus (input) and the body sway (output). Sway responses are averaged across all subjects’ postural platform stimulation sequence repetitions. The input follows a pseudo-random ternary signal profile known as PRTS (Peterka, 2002; Davies, 1970) (Figures 1A,B). This means that the signal alternates between three possible speed levels: zero (no movement), a positive speed (+s), and a negative speed (−s). The value of s is chosen carefully to ensure that the resulting movement reaches a specific range—typically measured in position changes, such as a tilt of 1° from peak to peak. In simpler terms, the movement follows a structured but unpredictable pattern, switching between these speed levels in a way that appears random, but is designed to test specific responses. A commonly used version of this PRTS profile is illustrated in Figure 1A.

The PRTS has a unique power spectrum, meaning that when analyzed in terms of its frequency content, it displays distinct peaks at certain frequencies. These peaks are separated by zones where no power is present, meaning that some frequency components are completely absent from the signal. This pattern, shown in Figure 1B, is important because it influences how the system being tested will respond to different frequency components of the input. The spectra of the corresponding stimuli and body sway responses in space are computed using Fourier transforms. Finally, frequency response functions are computed as cross-power spectra *G_xy_(f)* divided by the stimulus power spectra *G_yy_(f):*


$$\frac{G_{xy}}{G_{yy}}$$

The values of the obtained transfer functions (Figure 1C) are averaged over bands of frequencies, with the resulting FRFs being represented by a vector of 11 complex values. As a result, the FRFs are defined and plotted with respect to the following frequency points.

f=[0.050.150.30.40.550.70.91.11.351.752.2], as shown in Figure 1D.

Such FRF definition, with averaging over groups of frequencies, is particularly significant in posture control research, where it has been widely adopted in the field. It was originally introduced in Peterka (2002) and has since influenced numerous studies that built upon its methodology (for example Assländer et al., 2020; Ketterer et al., 2024; Missen et al., 2024; Assländer, 2015; Goodworth and Saavedra, 2021). One key methodological decision in FRF analysis is the grouping of frequencies for averaging. This practice was initially motivated by the need to display the FRF on a logarithmic scale (Goodworth and Saavedra, 2021). On such a scale, the higher-frequency bands naturally contain more data points than the lower-frequency bands. However, these high-frequency data points often have a lower signal-to-noise ratio, meaning they are more affected by random fluctuations in the data. To address this issue, researchers averaged more adjacent high-frequency points than low-frequency points. This equalizes the spacing of the FRFs when plotted on a logarithmic scale, making it easier to interpret trends across different frequencies. Additionally, this approach maintains similar confidence intervals across all frequency bands, ensuring that the reliability of the FRF estimates remains consistent (Goodworth and Saavedra, 2021; Goodworth and Peterka, 2009; Otnes and Enochson, 1972). This method also implies that high-frequency components of the response are considered less important within the experimental scope. This assumption is reasonable in many posture control experiments. For example, when a subject stands on a tilting support surface, the body’s postural control system behaves like a low-pass filter—meaning it reacts strongly to slow tilts, but does not respond as much to rapid, high-frequency oscillations. This low-pass characteristic is well-documented in studies on postural control and balance mechanisms (Peterka, 2002; Van Der Kooij and Peterka, 2011).

### 2.2 Experimental setup

The body sway responses used in this study were obtained from three groups of human subjects. The overall setup and detailed clinical inclusion criteria for the IPD and PSP groups are described in detail in Kammermeier et al. (2018), Kammermeier et al. (2024), and Lippi et al. (2023). Each group includes elderly subjects, among which were 17 healthy subjects, 11 advanced-stage IPD patients with frequent falls at least once a month and 17 ambulatory PSP patients. All but one PSP patients were also participants of the PROSPERA study (prematurely ended, randomized double-blinded Rasagiline in PSP, EudraCT number 2008–007520-26). All PSP patients were “Clinical Probable PSP” according to the NINDS-SPSP criteria (Respondek et al., 2013) valid at the time of study inclusion, subsequently refined and replaced by the Movement Disorders Society criteria of 2017 (Höglinger et al., 2017). All participants gave written informed consent, and data was anonymized at study inclusion following the Helsinki Declaration and the local ethics committee (decision 142/04; Ethikkommission der Medizinischen Fakultät der LMU). Patients wore their everyday clothing including shoes and were under their regular medication including prescribed dopaminergic medication in “ON,” to reflect conditions in which most falls occur in the clinical context, rather than creating an artificial barefoot and medication “OFF” condition, which would not reflect practical occurrence of the investigated falling problem.

**Figure 2:**
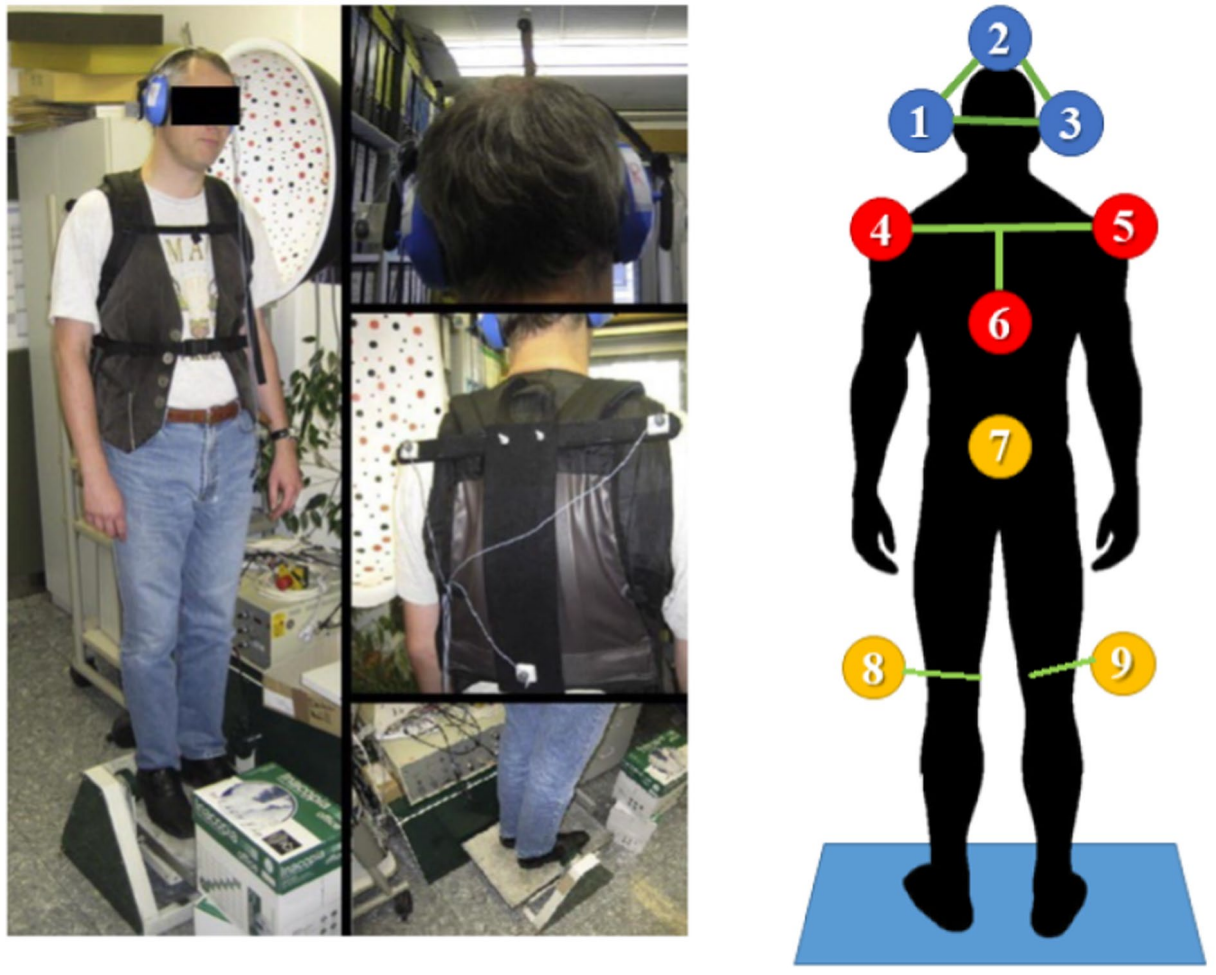
Left: The experimental setup. The test subject stands on a platform, tilting in the sagittal plane around the ankle. The subject’s feet were placed within marked positions, with the heels together and the tips spread 15° apart, while the arms hung loosely by the sides. The tilt axis of the platform was ensured to act through the anatomical upper ankle joint of the subject. Right: 3D ultrasound position markers (Zebris system) placement. Head (1, 2, 3), upper chest (4, 5, 6), hip (7), and lateral femoral epicondyles (8, 9). Pictures from (3). Patients were in their everyday clothing including shoes and on regular medication in dopaminergic “ON,” to reflect conditions in which most falls occur.

All subjects were placed on a remotely controlled platform (Toennis), as shown in Figure 2. The platform produced a front-to-back tilt oriented around the ankle joint axis by the mechanical setup of the platform, which is set up to rotate the surface of the shoes under the axis through the upper ankle joint (see Figure 2). The stimulation paradigm involved a resting condition and small-angle (0.5° and 1°) rotational disturbances with a pseudorandom PRTS profile for 60 s; each maximum angular displacement was tested in eyes-open EO and eyes-closed EC conditions.

3D motion capture was performed with ultrasound receivers (CMS20 Zebris 3D ultrasound motion capture system, Zebris GmbH, Isny im Allgäu, Germany).^1^ This is a standard set up for tracking the dorsal spine and body segments in posture control experiments (Fölsch et al., 2012; Malmström et al., 2003; Li et al., 2016). Markers were placed on the subjects, visible from behind, to track the head, upper trunk, hip, and knee position during the experiments, as shown in Figure 2. The ultrasound receiver CMS20S was placed 1 m behind subjects on the platform at roughly head level to get vision of all markers. The Zebris software was used to record the 3D positions.

**Figure 3:**
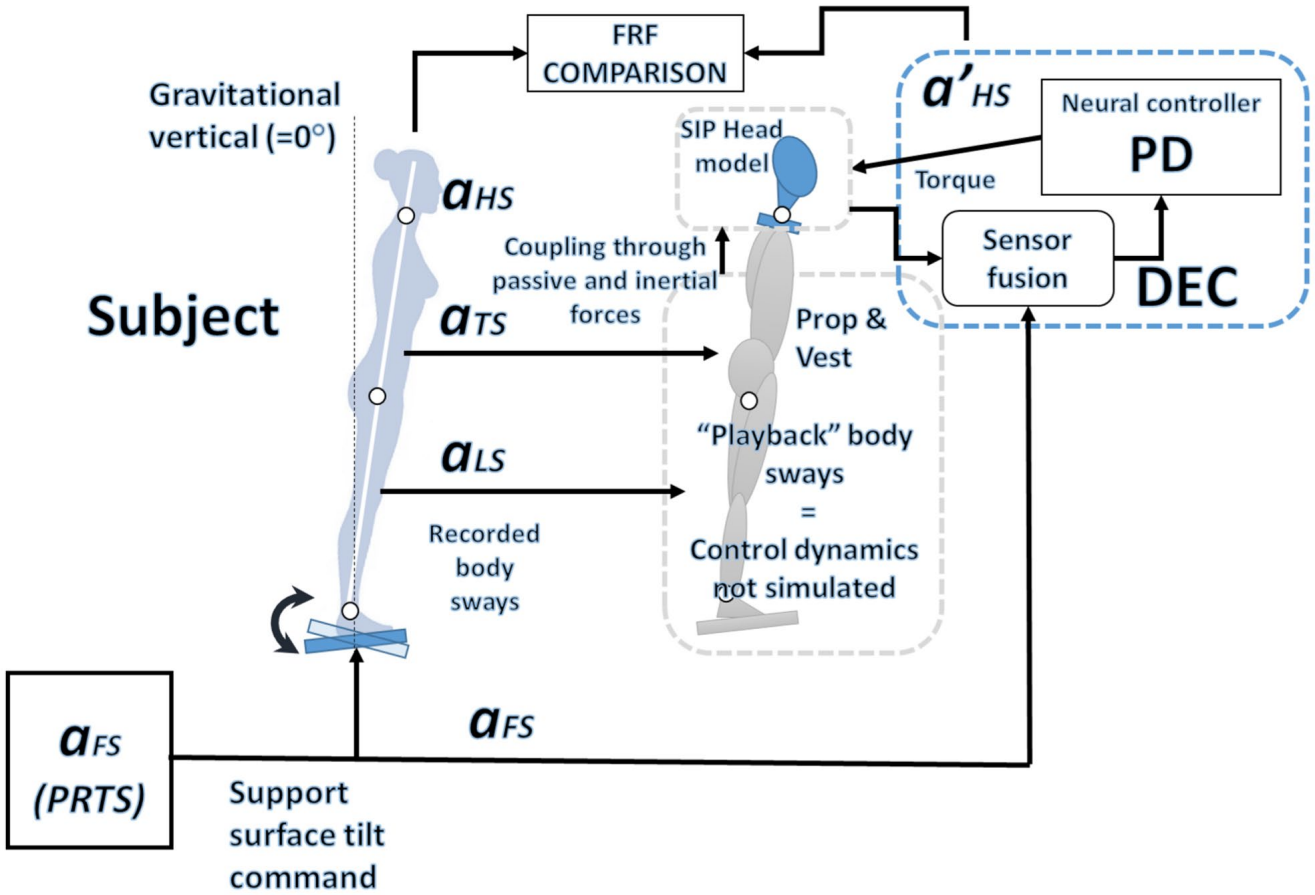
Overview of the simulation system: The observed oscillations in the legs and trunk, along with the predefined PRTS input profile, serve as inputs to model the motion of the neck.

The sampling rate was dynamic between 80 and 200 Hz depending on momentarily detectable markers and was resampled offline to 100 Hz using Matlab *Resamp* function. The sway of the body segments was computed using trigonometry, e.g., computing the inclination of the triangle defined by the markers around the head (1, 2, 3 in Figure 3). Considering a maximum error of 0.5 mm (Zebris, 2024) and a height of the marker triangle of 25 cm ca. the precision on HS was around 0.002°, enough to track head motion properly.

The final dataset included the platform motion track and the position signals of three markers for the head, three for the chest, one for the lower spine, and one each above the knees, each along the time domain.

### 2.3 Head posture control model

The model from Lippi et al. (2023) was used to fit the data. Specifically, in Lippi et al. (2023), some model variations were tested on healthy subjects; the model that produced the best fit was used here. The control model is based on the *disturbance identification and compensation* (DEC) principle (Mergner, 2010) and its implementation as a modular control system (Hettich et al., 2013; Hettich et al., 2014; Lippi and Mergner, 2017; Lippi, 2018). For a general and extensive description of the control system (see Lippi and Mergner, 2017). The DEC is based on the hypothesis of a servo controller for body position (Merton, 1953), complemented by the estimation and compensation of external disturbances based on sensory input (Mergner et al., 1997; Mergner and Rosemeier, 1998). The servo controller is implemented as a PD, proportional derivative controller. The compensation, which generally includes support surface tilt and acceleration, gravity, and external push, is considered only for gravity and support surface tilt. Such compensation is a feed-forward compensation of disturbances based on sensory input that allows us to estimate the disturbance itself (Bleisteiner et al., 1961). The DEC controller is mainly used to predict steady-state responses, unlike other models oriented to transient responses (Allum and Honegger, 1992; Küng et al., 2009).

The control equations regulating the action of the servo controller for the neck are:


(1) $$\mathrm{Active Torque}\;\tau_{act}=\left(K_{P}+K_{D}\frac{d}{dt}\right)\;\left(\varepsilon +\hat{G}\right)$$

(2) $$\mathrm{Passive Torque}\;\tau_{Pass}=\left(K_{PP}+K_{PD}\frac{d}{dt}\right)\;\left(\alpha_{HT}\right)$$

(3) $$\mathrm{Gravity Compensation}\;\hat{G}=K_{G}\alpha_{HS}$$

(4) $$\mathrm{Support Surface Tilt Compensation}\;{\hat{\alpha }}_{HS}=\alpha_{HS}+\alpha_{FS}-{\hat{\alpha }}_{FS}$$

Where $K_{P}$ and $\;K_{D}$ are the coefficients of the PD controller, ε is the error on the controlled variable that can be the estimated head in space position ${\hat{\alpha }}_{HS}$ from Equation 4 or $\alpha_{HT}$ , the angle between head and trunk, and $\hat{G}$ is the estimated gravity torque from Equation 3. The value $\hat{G}$ is expressed as an angle equivalent in order to be summed to ε in Equation 1. This means that the gravity estimation is divided by the mass of the head multiplied by the height of its center of mass and the gravity constant g. this is all considered in the parameter $K_{G}$ that, hence, is a unitless gain. Expressing the disturbance as an additional input for the PD exploits the derivative as an anticipation effect. In robotics applications, the compensation can have its own PD parameter to allow for fine control (Ott et al., 2016); here, only a singular PD is used, in accordance with previous works, where the DEC is used to model human responses (e.g., in Hettich et al., 2014; Mergner et al., 2009). $K_{PP}$ and $\;K_{PD}$ are the passive stiffness and damping associated with the neck. $\alpha_{HS}$ in Equations 3, 4 is the head-in-space angle (with respect to the gravitational vertical) that here is assumed to come from the vestibular system without any modeled noise. $K_{G}$ is a coefficient associated with gravity compensation. Usually, in humans, it is slightly under-compensated, considering the additional torque produced by the servo loop.


(5) $$\vartheta_{FS}\left(\alpha \right)=\{\begin{array}{cc} \alpha +\theta_{FS} & \alpha <-\theta_{FS}\\ 0 & -\theta_{G}<\alpha <\theta_{FS}\;\\ \alpha -\theta_{FS} & \alpha >\theta_{FS} \end{array}$$

The estimation of support surface tilt is affected by a nonlinearity reflected in the estimated ${\hat{\alpha }}_{HS}$ in Equation 4. The foot-in-space estimation is performed by fusing the vestibular angular velocity signal with the proprioception of all the joints from the head to the ankle; a nonlinear function $\vartheta_{FS}\left(\;\right)$ is defined as.

The threshold $\theta_{FS}$ is then applied to the resulting velocity signal. Here, as the proprioception and vestibular signals are modeled as ideal, the $\alpha_{FS}$ known from the experiment design (§2.2) is used to produce the following estimation


(6) $${\hat{\alpha }}_{FS}=\int_{0}^{t_{1}}\vartheta_{FS}\left(\frac{d}{dt}\alpha_{FS}\right)d\tau$$

Where $t_{1}$ is the current time and the initial condition of the estimator is assumed to be ${\hat{\alpha }}_{FS}=0$ . Again, for the assumption of ideal proprioceptive signals, the error $\alpha_{FS}-{\hat{\alpha }}_{FS}$ is propagated directly to ${\hat{\alpha }}_{HS}$ , leading to Equation 4. The nonlinearity $\vartheta_{FS}\left(\;\right)$ explains that smaller stimuli are associated with larger gains in the responses (Hettich et al., 2011). Although introducing an error in tracking body sway prevents asymptotic stability, the dead band does not make the system unstable; in fact, it has been demonstrated that the system is Lyapunov stable (Lippi and Molinari, 2020).

A lumped delay Δt representing all the delays in the loop affects the active control (it is in series with the PD). The sources of the delay are the sensory input and the motor control. For the control of the ankle, they are usually estimated to be between 80 and 200 ms, depending on the subject and the test conditions (Antritter et al., 2014; Li et al., 2012; Molnar et al., 2021). In general, the delay of peripheral body joints is expected to be larger than the one associated with joints closer to the brain, e.g., the delay in the control loop of the hip is smaller than the one of the ankle: 70 ms and 180 ms, respectively, in Hettich et al. (2011) and Antritter et al. (2014). This predicts that the delay in the neck control loop will be smaller. An overview of the simulated system is shown in Figure 3.

The dynamic of the head is simulated as a single inverted pendulum (SIP) characterized by the weight and the moment of inertia of the head, on which the active toques $\tau_{act}$ and $\tau_{Pass}$ from (1) and (2). The translation produces a further effect due to the sway of the legs and the trunk, resulting in the following dynamic system, where the small angle approximations sinα≈α and cosα=1 are applied:


(7) $$\{\begin{array}{ll} {\ddot{\alpha }}_{HS}= & \left(\tau_{act}+\tau_{pass}+G+T_{acc}\right)/J_{H}\\ G= & m_{H}gh_{H}\;\alpha_{HS}\\ T_{acc}= & \;\left({\ddot{\alpha }}_{LS}l_{L}+{\ddot{\alpha }}_{TS}l_{T}\right)\;h_{H}m_{H} \end{array}$$

**Figure 4:**
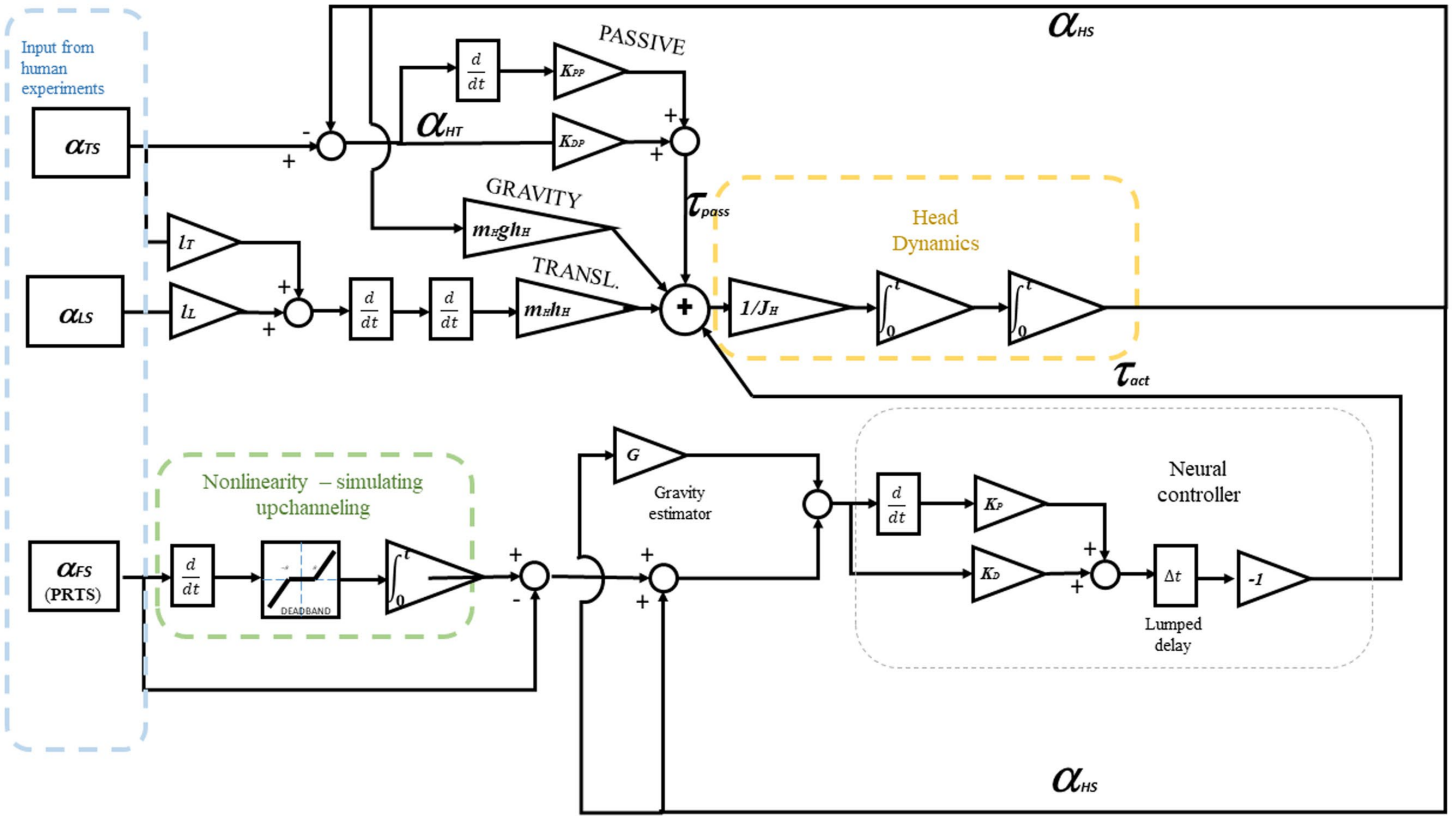
The Dynamic system from Equations 1–7. Here, the controlled variable is the head-in-space angle α_HS_. To control the head-to-trunk angle α_HT_, such a variable should be provided as input for the neural controller instead of α_HS_. The model includes head dynamics to evaluate the parameters relative to the control of the neck and uses the kinematics of the trunk and the legs as an input, a “playback” as shown in Figure 3. The lower part of the schema represents a sensor fusion process. As the reference is assumed to be 0°, the controlled variable equals the error *ε* in Equation 1. The recorded body sway for the trunk and legs is used as input. In contrast, the recorded head sway is used outside the simulation to optimize the parameters and evaluate the result.

Where $J_{H}$ is the head moment of inertia, $m_{H}$ is the head mass, $h_{H}$ e is the height of the head center of mass, and $g=9.81\;m/s^{2}$ is the gravity acceleration constant. $l_{L}$ and $l_{T}$ are the lengths of the trunk and leg segments, respectively. A standard set of anthropometric parameters (Winter, 2009; De Leva, 1996) is used in all the simulations with no specific adaptation to the single subject. Anthropometric parameters are reported in Table 1. The full dynamic system is shown in detail in Figure 4.

**Table 1:** Anthropometric parameters used in the posture control model.

Parameter	Symbol	Value
Head moment of inertia	*J_H_*	0.4797 kg/m
Leg length (ankle to hip)	*l_L_*	0.8543 m
Trunk length (hip to neck)	*l_T_*	0.5011 m
Head COM height	*h_H_*	0.2053 m
Head mass	*m_H_*	4.5 kg

### 2.4 Testing significant differences between FRFs

The differences between the groups’ averages are tested with a bootstrap method specifically proposed to test differences between groups of FRFs (Lippi, 2025a; Lippi, 2025b; Lippi, 2024). In detail, the confidence intervals for continuous functions (Lenhoff et al., 1999) are defined in the time domain. This is possible because the FRF is a transfer function, so its Fourier transform is the impulsive response of the system (real function). The method to obtain the confidence bands is discussed in detail in Lippi (2025a). Since the tests involve unpaired samples, two different groups of subjects are tested under the same conditions. Specifically, the bootstrap hypothesis test was conducted by repeatedly resampling the available data with replacement to generate an empirical distribution of differences between group averages. This approach does not assume normality and is well-suited for cases where the data distribution is unknown or irregular. The procedure follows these steps:

*Resampling:* each group is resampled independently, with replacement, to generate new synthetic datasets of the same size as the original samples.

*Computing the test statistic:* the difference between the means of the two groups is calculated for each resampled dataset. The test value used is the difference between the means normalized by the variance of all the samples in time domain.

*Building confidence bands:* the repeated resampling process generates a distribution of possible mean differences at each point in time. The confidence bands are then computed by selecting the percentiles corresponding to the desired confidence level (e.g., 95%). This results in upper and lower bounds that indicate the range within which the true mean difference is expected to lie.

*Testing the null hypothesis:* the confidence bands are compared against the horizontal axis, representing a zero difference between means. If the bands do not include this zero line over a significant portion of the domain, the null hypothesis (that there is no difference between the groups) is rejected.

The residuals are analyzed to visualize the results in the frequency domain. Residuals are defined as the differences between the confidence bands and the part of the tested sample that exceeds them. Once the confidence bands are constructed in the time domain, the residuals can be transformed to the frequency domain using the Fourier Transform. This process reveals how variability is distributed across different frequencies. Such representation provides a qualitative insight into whether the differences are localized at particular frequencies or spread across the spectrum, which is used after the test rejects the null hypothesis.

A library of functions to implement FRFs statistics and the specific tests with unpaired samples is available online (Lippi, 2024).

### 2.5 Model parameters identification

The identified parameters are *K_P_, K_D_, K_PP_, K_PD_, K_G_*, the threshold, and the lumped delay ∆*_t_*. The delay in the loop is important for shaping perception and action; for example, it is observed that an added delay of about 70–80 ms can already be perceived (Morice et al., 2008) and affect performance in tracking tasks (Lippi et al., 2010). Hence, it is reasonably one of the parameters characterizing the performance in posture control, as suggested by Li et al. (2012), Molnar et al. (2021), and Van Der Kooij et al. (1999), Lockhart and Ting (2007), and Vette et al. (2010). Delays are also a determining factor in the different performances of young and elderly subjects (Qu et al., 2009; Davidson et al., 2011). *Other parameters are feedback gains. Specifically, the effect on the body with the support surface rotation is due to the passive stiffness and the delays in the loops producing the active torque. The gain K_G_* applied on gravity feedback represents the (small) under-compensation of gravity observed in subjects. The body sway responses observed in experiments are nonlinear, and such nonlinear responses can be reproduced by the effect of the threshold (Mergner, 2010).

The fit is performed on the across-subject FRF response of each group of samples as in Assländer and Peterka (2014) and Pasma et al. (2018). This is done because, in the present work, the focus of the study is the difference between groups of FRFs associated with different conditions, difference that has been assessed with the test described in the previous subsection §2.4. The assumption is that group-averaged responses can effectively capture the central sensorimotor strategy, as they represent a cohort’s “typical” behavior. Using the group average as the fitting target provides a robust representation of the underlying control mechanisms. This approach reduces the influence of inter-subject variability and is expected to produce parameters that are representative of the group’s postural control strategy. This is similar to what is done in general in system identification where averaging multiple trials enhances the signal-to-noise ratio and yields more reliable parameter estimates by filtering out idiosyncratic fluctuations (Ljung and Ljung, 1999).

The model fits the data using a numeric research algorithm implemented by the Matlab function *fminsearch* that allows system simulation at each step. The objective function to be minimized is the difference between the FRF from the experimental trial and the one produced by the simulation (the *score* value in Table 2).

**Table 2:** Model parameters fit the average response of each group.

Group	Eyes	*K_PP_*	*K_DP_*	*K_P_*	*K_D_*	*∆_T_*	*K_G_*	*θ*_FS_	Score
CO	Closed	37.591	8.4535	0.004094	2.8939	0.039358	0.10619	0.02226	0.76525
CO	Open	43.029	7.5394	0.53982	0.96498	0.02217	0.095583	0.12776	0.87411
IPD	Closed	38.852	4.1253	5.7809	11.365	0.000673	0.018112	0.007753	0.64086
IPD	Open	15.499	1.9855	6.5599	5.3434	0.001211	0.020482	0.004569	0.86278
PSP	Closed	224.94	1.511	4.0473	10.459	0.00288	0.03196	0.000332	4.3685
PSP	Open	32.136	4.6341	9.0265	0.41382	0.11315	0.01004	0.00016	0.94434

The value Score represents the difference between the target FRF and the FRF produced by the simulation.

## 3 Results

### 3.1 Body sway responses

**Figure 5:**
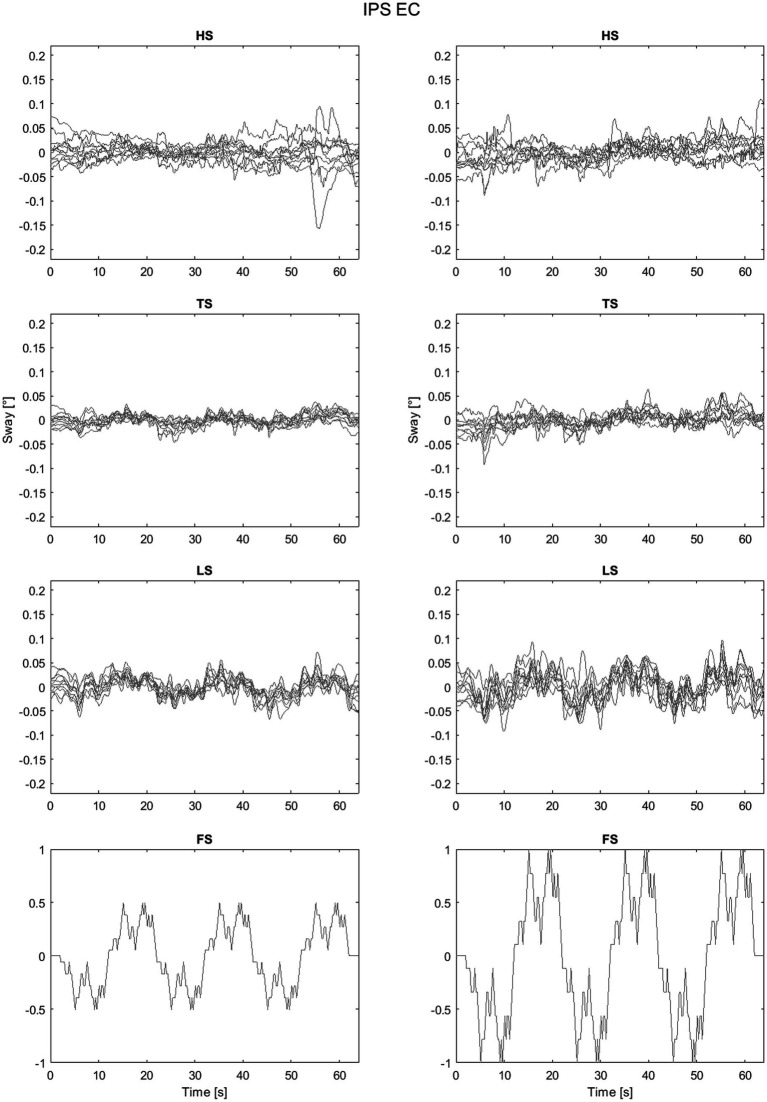
ISP body segments sway responses to 0.5° (left) and 1° peak-to-peak amplitude.

**Figure 6:**
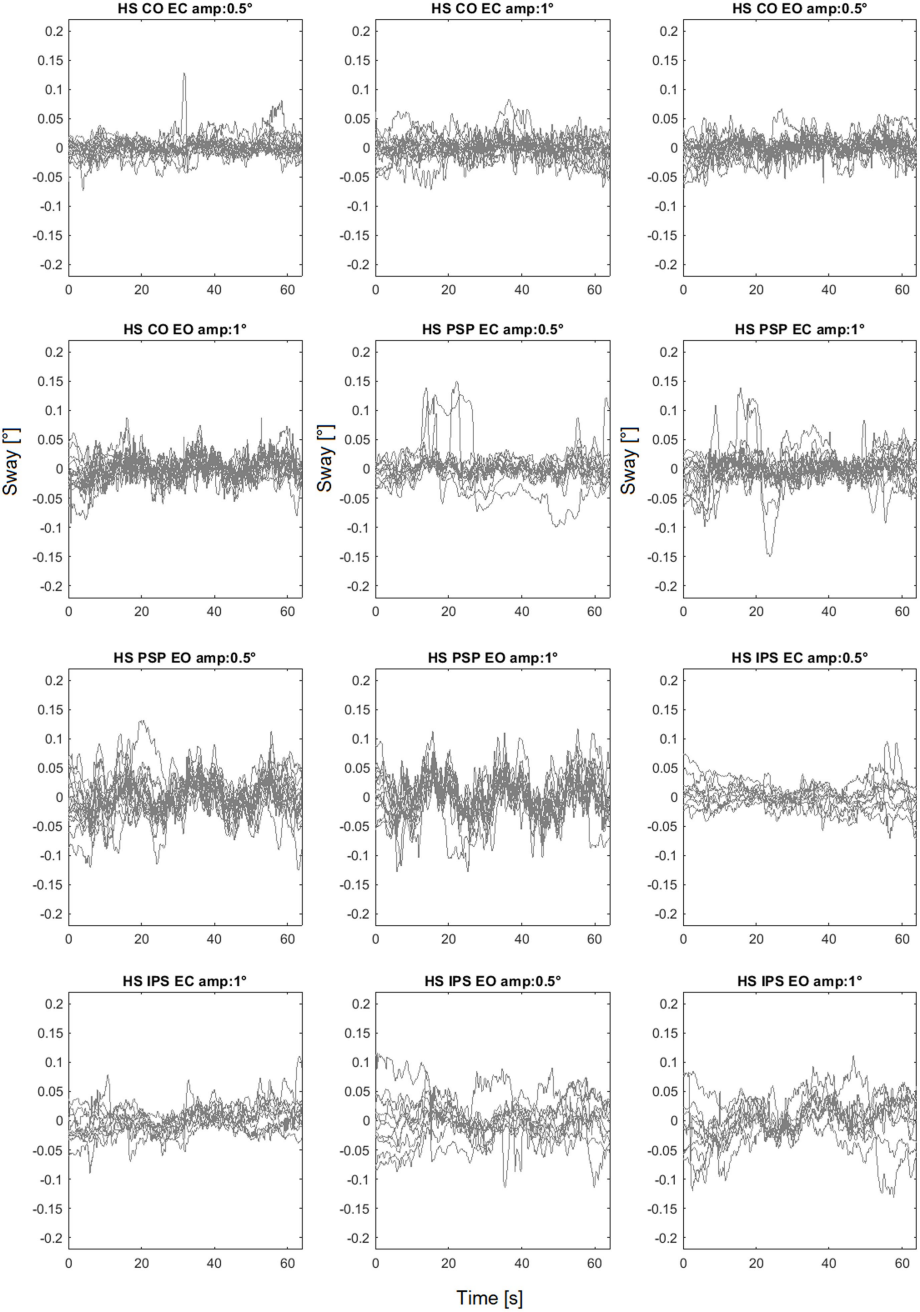
Head sway (head in space - HS) responses to different amplitudes. CO identifies the control group.

As an example of the full dataset obtained by the experiments the sway trajectories for head, trunk and legs for PSP patients with eyes closed are shown in Figure 5 All the head-in-space (HS) sway responses are shown in Figure 6. The plots provide an idea of the variability of the subjects’ responses within groups. Figure 6 exhibits the expected nonlinear response in that the difference between the LS response is relatively small compared to the proportion of the two peak-to-peak amplitudes (i.e., one twice the other) as expected from previous experiments (Mergner et al., 2009; Mergner et al., 2003). In some trials HS exhibits adjustments, i.e., movements in the order of 0.1° degrees that appear to change the offset of head position.

### 3.2 Comparison of the average responses

**Figure 7:**
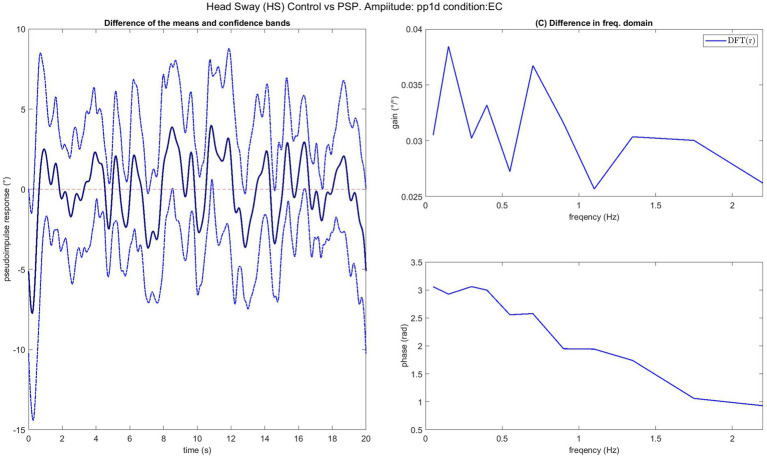
Bootstrap test comparing the average FRF of the control group and PSP for stimulus amplitude 1°. On the left is the difference between the group averages of *pseudo-impulsive responses* (PIR) of the two groups with 95% confidence bands. As the x-axis (representing a null difference between the group’s averages) is outside of the confidence bands, the two groups’ averages are assumed to be significantly (*p* < 0.05) different. On the right, the FRF of the residual, i.e., the difference between the bands and the x-axis, provides a view in the frequency domain of the difference between the two groups.

**Figure 8:**
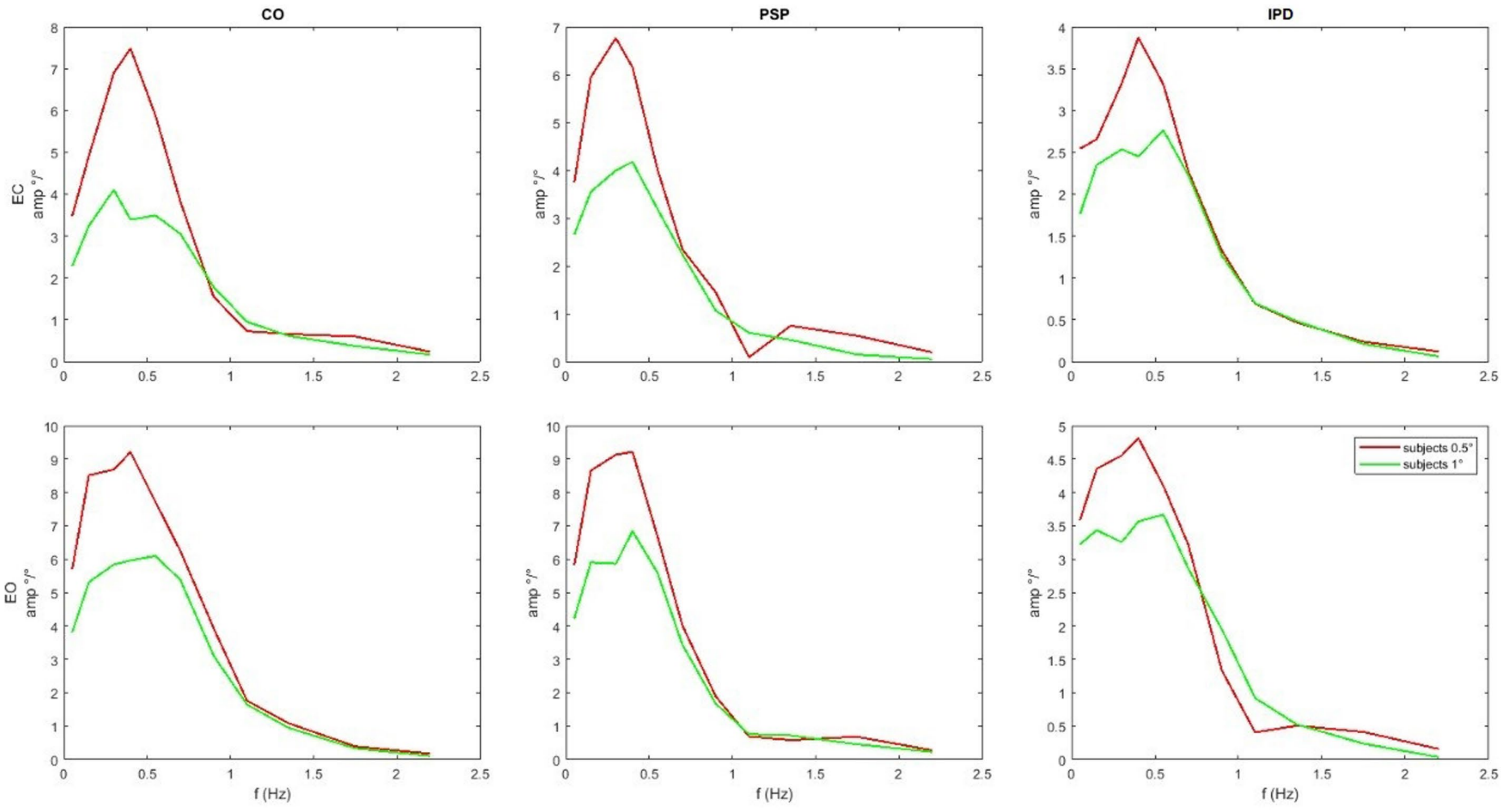
Average module of the COM sway responses. Notice that the scales of the graphs are different, and although exhibiting a similar low-pass profile, IPD is associated with smaller gains than the control group and PSP. The COM sway is not predicted by the model, but it is provided as input shown in Figure 3.

**Figure 9:**
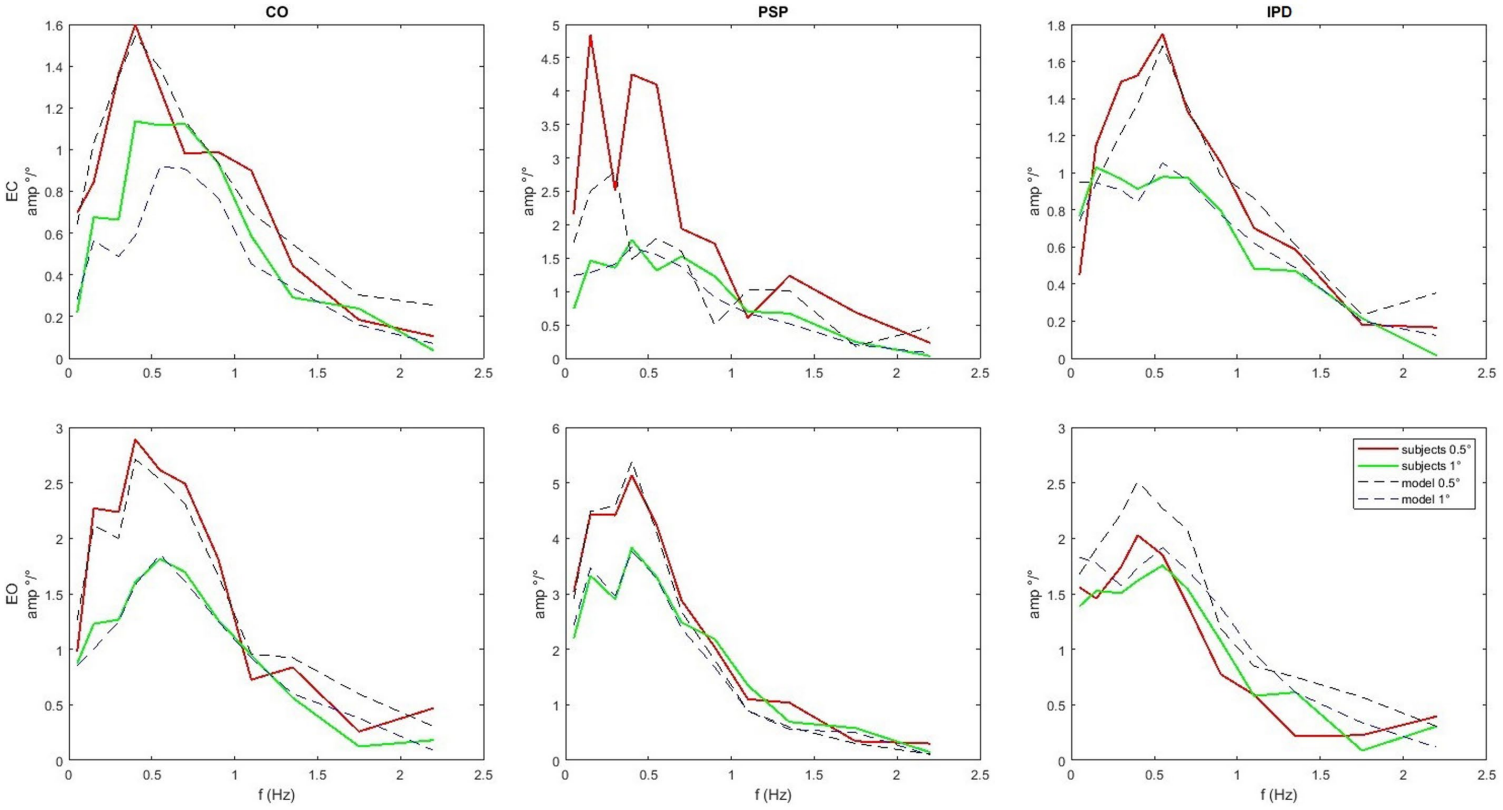
Average amplitude of the FRFs of head angle relative to the gravitational vertical for the sample set compared with the simulation’s results with the parameters from Table 2. Notice that the scale varies between subplots. PSP is generally associated with larger gains compared to the control. The response of the IPD subject has a larger gain with EC and smaller with EO compared to the control.

In Figure 7 one of the performed tests is displayed in an exemplary fashion (control vs. PSP patients at 0.5° eyes closed EC). The 95% confidence bands around the difference of the means of the groups do not include the lateral x-axis (no difference), resulting in a rejection of the null hypothesis, proposing that the average of the two groups is the same with *p <* 0.05 as discussed in the next paragraph. The COM responses are shown in Figure 8. The average head sway responses in the frequency domain for the groups under different conditions are reported in Figure 9. Figure 5 shows that, although exhibiting a similar low-pass profile in the FRF, IPD is associated with smaller average gains than the control group and PSP. The result for head control is different: in Figure 7, PSP is, in general, associated with larger gains compared to the control. The response of the IPD subject has a larger gain with EC and smaller with EO, compared to the control. This suggests a specific effect of the examined diseases on the control of the head, which cannot be trivially explained by the pattern of COM sway.

### 3.3 Significant differences between groups

The test specified in 2.4 was performed to compare the average FRFs of the three groups given the same conditions of visual input and signal amplitude. Significant differences were found between the average FRFs of the responses among the following groups:

For the COM sway:

1. Control vs. IPD 0.5° EC
2. Control vs. PSP 0.5° EO
3. Control vs. IPD 0.5° EO
4. Control vs. PSP 1° EO

And or the Head Sway:

1. Control vs. PSP 0.5° EO
2. Control vs. PSP 1° EC
3. Control vs. PSP 1° EO
4. Control vs. IPD 1° EO.

Also, the two groups of patients were compared, but no case showed a significant effect. PSP subjects were significantly different in head control in all the cases 5, 6, and 7, b3ut EC 0.5 pp. The residuals (see Figure 7) were distributed along all the frequencies without exhibiting any notable peak.

### 3.4 Model fit

The quality of the model fit is evaluated by comparing the experimental FRFs of the head-in-space responses with those simulated by the model using the optimized parameters listed in Table 2. In Figure 9, the average amplitude of the experimental FRFs across the relevant frequency bands is plotted alongside the simulation results. A close match between the experimental and simulated curves is observed, indicating that the model can capture the essential dynamics of head control across conditions and groups. In particular, the model accurately reproduces the low-pass behavior and the gain differences observed between the control, IPD, and PSP groups. The “Score” parameter in Table 2 quantifies the residual discrepancy between the target (experimental) FRF and the simulated FRF, with lower score values indicating a better fit. The score is computed based on the responses for both amplitudes as the same model is used to reproduce both. For instance, the relatively low score values for the control conditions suggest that the model well-represents the underlying control mechanisms in healthy subjects. Figure 9 shows that the simulation reproduces key features of the experimental frequency responses, such as the overall amplitude scaling and the frequency-specific response profiles.

## 4 Discussion

The presented model for postural head control in a steady state support surface oscillation paradigm allows for a detailed characterization of central postural control deficits in Progressive Supranuclear Palsy PSP and Idiopathic Parkinson’s Disease IPD, in comparison to healthy age-matched control subjects, based on averaged group data obtained from a clinical study.

Among the differences between the groups revealed by the statistical analysis were parameters obtained by fitting the average head responses. For both groups of patients, a larger passive stiffness *K_PP_* in EC trials suggested that the head tends to move together with the trunk in the eyes closed condition EC. With eyes open EO, the active proportional gain *K_P_* is relatively larger in all cases, suggesting that the head is directed closer to the space vertical by the visual contribution to the central integration (Kelly et al., 2008; Paulus et al., 1984). Since this held true for all of the investigated groups, findings support the notion of intact visual contribution to central postural control among PSP and IPD, despite the impaired supranuclear eye guidance among PSP.

In all cases, a larger gain was associated with smaller stimuli, but this effect was not as pronounced in IPD patients in the EO condition. Such nonlinear behavior has been properly predicted by adding the nonlinearity from Equation 5, 6. The threshold $\theta_{FS}$ was a notable model parameter that could assume distinct values as a segregation between different groups and conditions. The value of $\theta_{FS}$ was smaller for the patients’ groups than the control group, and larger for EC than EO in the control group. This suggests a paradoxically prompter response to external stimuli in difficult conditions (i.e., eyes closed and neurological impairment). This is consistent with previous findings (Kneis et al., 2020), indicating that patients with chemotherapy-induced neuropathy exhibited, on average, smaller body-sway/stimulus gains compared to healthy control, and in line with the general consensus of less error-tolerant postural control in peripheral sensory and/or central neurological impairment.

It should be noted that there are limitations in estimating the system’s delay. Firstly, phase lags caused by time delays and those introduced by system dynamics (e.g., low-pass filters or poles) can produce similar effects in frequency-domain models, particularly when the input lacks high-frequency excitation. This is a well-recognized limitation in system identification, where such components can become practically indistinguishable (Van Den Hof and Schrama, 1995). Specifically, in posture control experiments, the identified lump delay integrated different sources of sensory and motor delays. The identified delay may be shorter than the expected neural delay as low pass components of the model account for the phase-lag exhibited by the system as in (Van Der Kooij and Peterka, 2011), or be larger than anticipated, reflecting delays due to upper cognitive functions involved in more demanding tasks such as balancing on a rocking board as presented in Molnar et al. (2021) and Molnar and Insperger (2022). A further thing to consider in the present work is that when the delay is very small the identified system can be under-constrained given the reference FRF, in that the active and passive feedback gains are practically indistinguishable in the absence of delay and with the trunk maintaining an upright stance as they both act producing a torque that pulls the head to the upright position. These limitations should be considered before interpreting the lumped delay as a measure of the neural delay. Nevertheless, the delay as a parameter is useful in characterizing the response of the different groups under different conditions and differentiating between them.

Considering the perspective of using the model to perform tests on patients in future clinical settings, efficient and rapid estimation of the parameters from patients’ trials can be obtained using neural networks (Ljung et al., 2020), as demonstrated in preliminary results for posture control (Lippi et al., 2020). Furthermore, modeling neck behavior could be useful when the DEC is applied to an assistive device for everyday use (Lippi and Mergner, 2020), especially if the device supports head movements like an active version of Garosi et al. (2022).

Future work will exploit the possibility of retrieving model parameters for individual trials from single patients. This can be performed with patients with a known condition to monitor the progression of the disease (i.e., testing different time points in the disease). System parameters can be used as a tool for diagnosis through the application of pattern recognition techniques (for example, in Krafczyk et al., 2006). Modeling the posture control task could lead to the possibility of designing interventions on the system to mitigate the effects of the respective diseases, for example, predicting the effect of known drugs on the specific condition, as presented in Glasauer and Rössert (2008).

## 5 Summary and outlook

The purview of this study was to establish a model of head stabilization control, separating characteristic features of PSP and IPD disease versus healthy controls based on averaged group data. This model illuminates differential central neural strategies and deficits between disease entities and may aid in refining robotics stability.

Future work will focus on adapting the present averaged group model to interpret variance in individual subject trials with known or unknown disease status. Adaptation to individual trials can be used to describe alterations of parameters during long-term progression of neurodegenerative disease and possible influence of therapeutic attempts, possibly discovering new subsets of parameters affected particularly during disease progression. Implementation should allow for immediate data interpretation after individual trials in conjunction with other studies, e.g., imaging and biobanking.

## Data Availability

The original contributions presented in the study are included in the article/supplementary material, further inquiries can be directed to the corresponding author.

## Footnotes

^1^ www.zebris.de
